# Changes in Economic Hardships Arising During the COVID-19 Pandemic: Differences by Nativity and Race

**DOI:** 10.1007/s10903-022-01410-z

**Published:** 2022-11-05

**Authors:** Allison Bovell-Ammon, Stephanie Ettinger de Cuba, Félice Lê-Scherban, Lindsey Rateau, Timothy Heeren, Cerlyn Cantave, Kaye-Alese Green, Deborah A. Frank, Diana Cutts, Eduardo Ochoa, Megan Sandel

**Affiliations:** 1 Boston Medical Center, 801 Albany Street, Boston, MA 02119 USA; 2 Boston University School of Public Health, Boston University School of Medicine, Boston, MA USA; 3 Drexel University Dornsife School of Public Health, Philadelphia, PA USA; 4 School of Public Health, Biostatistics and Epidemiology Data Analytics Center, Boston University, Boston, MA USA; 5 Hennepin County Medical Center, Minneapolis, MN USA; 6 College of Medicine, University of Arkansas for Medical Sciences, Little Rock, AR USA

**Keywords:** COVID-19 pandemic, Food insecurity, Behind on rent, Families with young children, Immigrant families

## Abstract

Hardships in early childhood impact health. Few longitudinal studies have examined pandemic-related hardships among families with young children by race/ethnicity or nativity. We used prospective longitudinal data from 1,165 caregivers of children < 4 years surveyed in English and Spanish face-to-face in 5 urban hospitals 1/2018 to 3/2020 (pre-pandemic) and again by telephone 9/2020 to 3/2021 (during pandemic). Caregivers reported hardships (household food insecurity [HFI], child food insecurity [CFI]), behind on rent [BOR]) and maternal race/ethnicity and nativity. During the pandemic vs pre-pandemic, families with immigrant mothers had greater increases in HFI [aOR = 2.15 (CI 1.49–3.09)] than families with US-born mothers [aOR = 1.44 (CI 1.09–1.90)] and greater increases in BOR [families with immigrant mothers aOR = 4.09 (CI 2.78–6.01) vs. families with US-born mothers aOR = 2.19 (CI 1.68–2.85)]. CFI increases for all groups did not vary by nativity nor race/ethnicity. HFI and BOR increases during COVID were significantly greater in families with Latina mothers and those with immigrant mothers than other groups.

## Introduction

Early childhood is a critical window of development; even brief periods of hardship may have long-term health impacts. Before the COVID-19 pandemic, food insecurity and housing instability were identified as prevalent social determinants of health in the United States (US) and strongly associated with adverse health conditions across the lifespan, including early childhood [1, 2]. Studies conducted during the pandemic found that households with young children, Black and Latinx adults, and immigrants experienced higher rates of economic hardship than the general population, a phenomenon researchers have attributed to the persistent burdens of systemic racism and xenophobia [3–5].

Previous studies on pandemic-related hardships were predominantly cross-sectional and limited to respondents with English fluency and internet access [3, 4]. The current multilingual, longitudinal study compared changes in experiences of hardships from pre-pandemic to during the pandemic among families with young children by race, ethnicity, and nativity.

## Methods

Data were collected through a prospective longitudinal study of families surveyed face-to-face in English or Spanish pre-pandemic, between January 2018-March 2020, in emergency departments or primary care clinics in 5 sites (Boston, Baltimore, Minneapolis, Philadelphia, Little Rock). Families were recruited to participate in a study examining the effects of economic hardships and public program participation on the health of young children and their families. Eligibility criteria included state residency, primary caregiver, and child aged < 48 months. Eighty-six percent of children were publicly insured. Telephone follow-up surveys of previously interviewed caregivers were conducted September 2020-March 2021. Each research site received institutional review board approval.

There were 6,875 caregivers eligible for this study who were interviewed pre-pandemic. Of these caregivers, we interviewed 1,165 at follow-up. Average time between baseline and follow-up survey was 23.8 months. The follow-up cohort did not differ from the pre-pandemic baseline cohort in household income, marital status, Supplemental Nutrition Assistance Program (SNAP) participation, or caregiver or household employment. The proportion of Latina and immigrant mothers in the follow-up cohort was higher than baseline.

In both baseline and follow-up surveys, caregivers reported on past-year household food insecurity [HFI], child food insecurity [CFI] (using the U.S. Household Food Security Survey Module: Six-Item Short Form for HFI and 3 items from the child scale for CFI) and on whether they were behind on rent (BOR), a risk factor for housing instability, including eviction [2, 6]. Hardship variables were dichotomous.

We assessed changes for each respondent in hardships from before to during the pandemic (pandemic period). Bivariate comparisons utilized chi-square and t-tests or ANOVA for categorical and continuous variables, respectively. We used unadjusted and adjusted repeated measures logistic regression to estimate pre-pandemic to pandemic changes in hardships and included interaction terms to assess differences by self-reported nativity of the mother (U.S.-born, Immigrant) and maternal race/ethnicity (Latinx, Non-Latinx Black, Non-Latinx White, Non-Latinx Other/Multiple Races) (Interaction terms nativity*pandemic period and race/ethnicity*pandemic period respectively). Where significant interaction was found, nativity-specific or race/ethnicity-specific adjusted odds ratios were calculated from the interaction model. There was insufficient sample size to analyze Non-Latinx Other/Multiple Races separately. Models were adjusted for caregiver education, number of children in the household, child age, and any employment in the household based on prior work demonstrating associations with economic hardship [2].

## Results

**Fig. 1:**
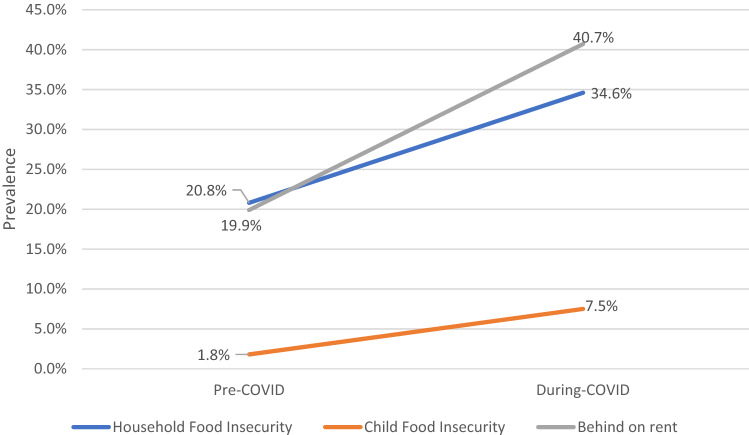
Trends in household and child food insecurity and behind on rent status before and during the COVID-19 pandemic among families with young children (n = 1,162) Source: Children’s HealthWatch data, 2018–2021

At intake, mean baseline age of children was 24.3(s.d.14) months. Demographics of mothers were 29% immigrant, 39% Latina, and 40% Non-Latinx Black. Overall prevalence of HFI increased from 20.8% pre-pandemic to 34.9% during the pandemic, and CFI from 1.8 to 7.4%. Overall prevalence of BOR doubled from 19.9 to 40.8% (Fig. 1).

Longitudinal adjusted analyses demonstrated disparate pandemic effects on hardships by nativity and race/ethnicity. Compared to pre-pandemic, families with U.S.-born mothers had 1.44 (95%CI 1.09, 1.90) times greater adjusted odds of HFI and 2.19 (95%CI 1.68, 2.85) times greater adjusted odds of BOR; families with immigrant mothers had 2.15 (95%CI 1.49, 3.09) times greater adjusted odds of HFI (interaction p = 0.03) and 4.09 (95%CI 2.78, 6.01) times greater adjusted odds of BOR (interaction p < 0.01) (Table 1, 2).

**Table 1:** Demographic characteristics of sample by food insecurity and behind on rent status at baseline

	Food Insecurity (Baseline) (N = 1165)			Behind on Rent (Baseline) (N = 1115*)		
Response	Household Food Secure	Household Food Insecure	p-value	Not behind on rent	Behind on Rent	p-value
	**925 (79.4%)**	**240 (20.6%)**		**899 (80.6%)**	**216 (19.4%)**	
Site, n (%)						
Baltimore, MD	99 (10.7%)	10 (4.2%)	< .001	90 (10.0%)	19 (8.8%)	< .001
Boston, MA	222 (24.0%)	84 (35.0%)		193 (21.5%)	79 (36.6%)	
Little Rock, AR	249 (26.9%)	73 (30.4%)		267 (29.7%)	52 (24.1%)	
Minneapolis, MN	102 (11.0%)	18 (7.5%)		92 (10.2%)	23 (10.6%)	
Philadelphia, PA	253 (27.4%)	55 (22.9%)		257 (28.6%)	43 (19.9%)	
Maternal nativity, n (%)						
US-born	668 (72.6%)	152 (63.6%)	< .001	626 (70.1%)	159 (73.6%)	0.31
Immigrant	252 (27.4%)	87 (36.4%)		267 (29.9%)	57 (26.4%)	
Child Age during pandemic, months						
N Mean (Std Dev) Median (25th, 75th)	925 46.7 (16.6) 48.6 (35, 59)	240 49.4 (15.6) 50.2 (39, 60)	0.02	899 47.1 (16.5) 48.92 (36, 59)	216 48.7 (16.5) 50.3 (37, 59)	0.27
Maternal race and ethnicity, n (%)						
Latinx	362 (40.0%)	84 (35.1%)	0.12	367 (41.7%)	60 (27.9%)	< .001
Black, non-Latinx	350 (38.7%)	105 (43.9%)		322 (36.6%)	114 (53.0%)	
White, non-Latinx	155 (17.1%)	34 (14.2%)		157 (17.8%)	23 (10.7%)	
Other race/ethnicity/ Multiple races	38 (4.2%)	16 (6.7%)		34 (3.9%)	18 (8.4%)	
Caregiver education at baseline, n (%)						
Some high school or less	134 (14.5%)	44 (18.4%)	0.23	136 (15.1%)	31 (14.5%)	0.67
High school graduate	334 (36.2%)	76 (31.8%)		322 (35.9%)	71 (33.2%)	
Technical school, some college, college graduate	455 (49.3%)	119 (49.8%)		440 (49.0%)	112 (52.3%)	
Maternal age at baseline, years						
N Mean (Std Dev) Median (25th, 75th)	908 30.4 (6.4) 30.0 (26, 35)	236 30.8 (6.4) 30.0 (26, 36)	0.36	880 30 (7) 30,(26, 35)	214 31 (6) 30 (27, 35)	0.15
Time between baseline and during pandemic survey, months						
N Mean (Std Dev) Median (25th, 75th)	891 23.6 (7.7) 24.2 (17, 31)	233 24.4 (7.7) 26.0 (18, 31)	0.21	864 23.6 (7.8) 24 (17, 31)	212 24.4 (7.7) 26 (18, 32)	0.14
Any household employment during pandemic, n (%)						
No	679 (76.9%)	194 (84.7%)	0.01	649 (76.0%)	180 (85.7%)	< .01
Yes	204 (23.1%)	35 (15.3%)		205 (24.0%)	30 (14.3%)	
Number of children in household during pandemic, n (%)						
N Mean (Std Dev) Median (25th, 75th)	876 3 (1) 2 (2, 3)	228 3 (1) 2 (2, 3)	0.30	848 3 (1) 2 (2, 3)	209 3 (1) 3 (2, 4)	0.13
SNAP participation during pandemic, n (%)						
Yes	442 (50.5%)	132 (57.9%)	.05	412 (48.6%)	127 (60.8%)	< .01
Household food insecurity during pandemic, n (%)						
Household food insecure	252 (28.7%)	134 (58.8%)	< .001	588 (69.2%)	103 (49.0%)	< .001
Child food insecurity during pandemic, n (%)						
Child food insecure	41 (4.7%)	40 (17.5%)	< .001	804 (95.1%)	174 (82.9%)	< .001
Behind on rent during pandemic						
Yes	332 (37.9%)	118 (52.0%)	< .001	304 (35.8%)	133 (64.3%)	< .001

^*^Sample size reflects participants with behind on rent response at baseline

**Table 2:** Associations between household food insecurity and behind on rent during pandemic compared to pre-pandemic baseline by caregiver nativity

	AOR (95% CI)
Household food insecurity	
Pandemic vs. Pre-Pandemic—US-born caregivers	1.44 (1.09, 1.90)
Pandemic vs. Pre-Pandemic—Immigrant caregivers	2.15 (1.49, 3.09)
Behind on rent	
Pandemic vs. Pre-Pandemic—US-born caregivers	2.19 (1.68, 2.85)
Pandemic vs. Pre-Pandemic—immigrant caregivers	4.09 (2.78, 6.01)

Nativity-specific AORs calculated from the interaction model

Interaction term, household food insecurity: Nativity*Pandemic period p-value = 0.03

Interaction term, behind on rent: Nativity*Pandemic period p-value = 0.001

Covariates: Education, number of children in the household, child age, any employment in the household, race/ethnicity

Families with Latina mothers (Latinx families) had 2.13 (95%CI 1.51, 3.01) times greater adjusted odds of HFI compared to pre-pandemic while those of other races/ethnicities who did not experience significant changes in HFI over time (interaction p = 0.04) (Table 3). Non-Latinx White families had 2.17 (95%CI 1.25, 3.78) times, Non-Latinx Black families had 2.08 (95%CI 1.52, 2.86) times, and Latinx families had 3.66 (95%CI 2.57, 5.20) times greater adjusted odds of BOR during the pandemic, compared to pre-pandemic (interaction p = 0.02) (Table 3). CFI increased for all groups, but the increase did not vary by race/ethnicity and nativity.

**Table 3:** Associations between household food insecurity and behind on rent during pandemic compared to pre-pandemic baseline by caregiver race/ethnicity

	AOR (95% CI)
Household food insecurity	
Pandemic vs. Pre-Pandemic—Non-Latinx White	1.25 (0.77, 2.02)
Pandemic vs. Pre-Pandemic—Latinx	2.13 (1.51, 3.01)
Pandemic vs. Pre-Pandemic—Non-Latinx Black	1.32 (0.95, 1.83)
Behind on rent	
Pandemic vs. Pre-Pandemic—Non-Latinx White	2.17 (1.25, 3.78)
Pandemic vs. Pre-Pandemic—Latinx	3.66 (2.57, 5.20)
Pandemic vs. Pre-Pandemic—Non-Latinx Black	2.08 (1.52, 2.86)

Race/ethnicity-specific AORs calculated from the interaction model

Interaction term: Race/ethnicity*Pandemic period p-value = 0.04

Covariates: Education, number of Children in the household, child age, any employment in the household, nativity

## Discussion

This study yields timely information on food and housing hardship among households with young children by race, ethnicity and nativity who are understudied in national datasets. We observed significant increases in pandemic-related HFI, CFI, and BOR across all groups; immigrant and Latinx families experienced the greatest increases in HFI and BOR. HFI and BOR are associated with negative health and developmental outcomes for young children [1, 2]. These longitudinal findings highlight the impact of the pandemic on families of young children with low incomes, with a disparate impact on immigrant and Latinx families.

While federal and state governments allocated trillions of dollars to relief efforts during the pandemic, the mechanisms for distribution of funding implicitly or explicitly excluded some families in need of assistance. Explicitly, many policies excluded some immigrants and implicitly, several barriers prevented families with low incomes—disproportionately families of color and immigrant families—from accessing benefits. For example, Economic Impact Payments (“stimulus checks”) under the Coronavirus Aid, Relief, and Economic Security (CARES) Act passed in March 2020 excluded nearly three million U.S. citizens and lawfully present immigrants in mixed immigration status families due to a Social Security number requirement for heads of households and their spouses. The second round of Economic Impact Payments passed in December 2020 extended eligibility to some mixed immigration status families, but exclusions persisted. Implicitly, the policy created barriers for over 12 million low-income households who are not required to file taxes because the payments’ delivery mechanism relied primarily on prior years’ tax filing to determine eligibility [7]. Another example was the increase in SNAP benefits for millions of families included in the Families First Coronavirus Response Act passed in March 2020. The initial structure of the policy change increasing benefits to the maximum amount meant those with the lowest incomes already at the maximum benefit amount did not see any increase until passage of subsequent relief packages, while pre-existing eligibility requirements meant that some otherwise eligible lawfully present immigrants who arrived in the U.S. within the past five years remained disqualified for the program [8]. In addition to structural barriers in relief policies, well-documented chilling effects of increasingly exclusionary immigration policies on families with any immigrant members, even if lawfully present, left many immigrants fearful of accessing benefits for which they and their children were eligible [9, 10]. More research is necessary to elucidate the impact of changing relief policies and programs over time on the outcomes observed in this study within and across racial/ethnic groups of varying nativity.

We acknowledge the limitations of this study. First this is a sentinel sample and, therefore, is not nationally representative, which limits generalizability. All outcomes in this study were self-reported and subject to shared method variance. The follow-up cohort had a greater proportion of immigrant and Latinx mothers than the baseline sample, but other demographic characteristics did not differ. Since we do not have information on the reasons for non-participation in the follow-up phone survey, we are unable to determine how attrition from the baseline may have affected results. Future research on factors such as moving during the pandemic, lack of access to phone service, homelessness, or other potential reasons for non-participation and their impact on hardships may be warranted. Despite these limitations, these longitudinal findings suggest the importance of scrutinizing the inequitable effects of the pandemic in the design of targeted pandemic relief policies.

Given the robust evidence of adverse effects of hardships on pediatric health, policy solutions to mitigate increases in economic hardships are necessary for all families of young children. Recent regulatory and legislative action to enhance immigrant access to benefits, including repeal of changes to the public charge rule and inclusion of mixed status families in the 2021 Economic Impact Payment, may reduce the disparities found in this study. However, formal exclusions from federal nutrition, housing, and cash benefits are ongoing and fear of deportation remains. Given that one-quarter of children in the US have an immigrant parent, permanent, inclusive, and equitable measures that reduce hardships are essential for optimal child and family well-being and the promotion of racial/ethnic health equity.

## New Contributions to the Literature

To our knowledge, this is the first study to analyze economic hardships experienced by families with young children during the COVID-19 pandemic by race/ethnicity and nativity. Given the importance of reducing food and housing hardships during this critical window of growth and development, this study contributes timely and important information for informing public policy decisions, especially those related to equitable economic recovery and reducing economic hardships among families with infants and toddlers.
